# Multimodal Image Reconstruction Using Supplementary Structural Information in Total Variation Regularization

**DOI:** 10.1007/s11220-014-0097-5

**Published:** 2014-08-21

**Authors:** Daniil Kazantsev, William R. B. Lionheart, Philip J. Withers, Peter D. Lee

**Affiliations:** 1 The Manchester X-ray Imaging Facility, School of Materials, The University of Manchester, Manchester, M13 9PL UK; 2 The Research Complex at Harwell, Rutherford Appleton Laboratory, Didcot, Oxfordshire OX11 0FA UK; 3 School of Mathematics, Alan Turing Building, The University of Manchester, Manchester, M13 9PL UK

**Keywords:** Hybrid modalities, Hybrid medical scanners, Structural prior, Anatomical prior, Image fusion, Positron emission tomography

## Abstract

In this paper, we propose an iterative reconstruction algorithm which uses available information from one dataset collected using one modality to increase the resolution and signal-to-noise ratio of one collected by another modality. The method operates on the structural information only which increases its suitability across various applications. Consequently, the main aim of this method is to exploit available supplementary data within the regularization framework. The source of primary and supplementary datasets can be acquired using complementary imaging modes where different types of information are obtained (e.g. in medical imaging: anatomical and functional). It is shown by extracting structural information from the supplementary image (direction of level sets) one can enhance the resolution of the other image. Notably, the method enhances edges that are common to both images while not suppressing features that show high contrast in the primary image alone. In our iterative algorithm we use available structural information within a modified total variation penalty term. We provide numerical experiments to show the advantages and feasibility of the proposed technique in comparison to other methods.

## Introduction

The problem of combining several images into a single one is an old problem of image fusion [1]. The challenging task is to transfer information from all data sets into a single domain which represents all the available data in the most complete way. The problem of fusing images can arise in many applications where data is acquired from different imaging systems or modalities. Recent advances in medical hybrid scanners have posed new challenges in data fusion between data sets representing different characteristics of the biological materials [2].

Functional imaging modalities, such as positron emission tomography (PET) or single photon emission computed tomography (SPECT) are used for diagnosing and monitoring oncological diseases. In medical hybrid scanners, the functional modalities are combined with anatomical imaging systems, such as X-ray computed tomography (CT) or magnetic resonance (MRI), to help in identifying the exact location of the decease. Complementary CT information is also used in PET and SPECT for attenuation correction or in some cases for the partial volume correction (PVC) which leads to improvement in resolution for functional images [3]. The measured data from hybrid scanners can be reconstructed separately and fused [4] or PVC corrected, or alternatively, the information on anatomical features can be embedded directly into reconstruction process by means of a priori information [5].

In this paper, we propose an algorithm which uses available information from one data set to increase the resolution and signal-to-noise (SNR) ratio of another one. The method operates on the structural information only which increases its suitability across various applications [6–10].

In [10], we used a diffusion tensor framework to build a combined tensor which exhibits the local structural properties for two datasets simultaneously. The modified diffusion tensor was used in the regularization framework to reconstruct SPECT measurements given the reconstructed MR image. The resolution of SPECT functional images was improved by the structural information from the referenced MR images. However the feasibility of the proposed technique was limited by the number of parameters needing definition. In this paper, we present a novel, yet more flexible and easy-to-use method, which has the same objective as the algorithm in [10].

The core of the new method is based on the total variation (TV) semi-norm, which has proven to be a successful tool for image recovery over the past few decades [11]. The apparent drawback of the TV semi-norm in favouring piecewise constant solutions to smooth solutions, although by considering higher order regularization terms this shortcoming can be suppressed [12–16]. The two-step algorithm proposed to solve the problem [12]. In the first step, one needs to smooth the vector field of the noisy image and then find a surface which fits the smoothed vector field (the second step). Subsequently, various enhancements and modifications of this method have been presented [13–16].

In this work, we are not concerned with the step of smoothing normals [12–15] or tangent vectors [16]; rather we investigate a situation where a supplementary vector field is available in the surface fitting step. We then modify the surface fitting step in such a way that additional information on edges can be easily integrated into the recovery process. Our aim is to encourage structural alignment of two images when gradient orientations tend to be parallel. On the other hand, non-parallel direction of level sets must be treated as a special case to avoid strong bias in the recovery process [10].

Our approach is tested on an image denoising and debluring problem and then applied to synthetic PET/CT reconstruction of a thorax. In these experiments, we consider the case when some image parts have common edges and some are structurally different. For image reconstruction experiment we introduce lesions into the synthetic functional phantom which are absent from the supplementary anatomical image. The main goal is to enhance the spatial resolution of functional images without loss of important features, such as introduced lesions. We compare the proposed method with another state-of-the-art method which uses supplementary information.

## Method

### Image Recovery by the Surface Fitting

For a given noisy image $\lambda _{0}(x,y) = \lambda (x,y) + \eta (x,y)$, which is defined on a two dimensional rectangular domain $\varOmega \subset {\mathbf{R}}^{2}$, one can find its noiseless representation $\lambda (x,y)$ recovered from noise $\eta (x,y)$ by minimizing the following cost function:

1 $$\begin{aligned} \min _{\lambda } \frac{\gamma }{2}\int _{\varOmega }(\lambda -\lambda _{0})^{2}\mathrm {d}\varOmega + \int _{\varOmega }|\nabla \lambda |_{\epsilon }\mathrm {d}\varOmega , \end{aligned}$$

where $\gamma$ is a regularization parameter to determine a trade-off between the data fidelity and TV semi-norm [11] respectively. The magnitude of the gradient $|\nabla \lambda |_{\epsilon } = \sqrt{\lambda _{x}^2 + \lambda _{y}^2 + \epsilon ^{2}}$ is calculated with a small constant $\epsilon$ to avoid instabilities in the uniform regions of $\lambda$. The minimization of (1) results in the noiseless piecewise constant approximation (cartoon or staircase effect) to $\lambda$ [11].

One can overcome the cartoon effect of the TV minimization (1) by considering a higher order regularization terms with a two-step minimization approach [12–16]. The first step is generally performed with the regularization of the vector field for $\lambda$, e.g. unit normal vector field [12]:

2 $$\begin{aligned} \min _{|\mathbf{n}(\lambda )| = 1} \frac{\delta }{2}\int _{\varOmega }(\mathbf{n}(\lambda )-\mathbf{n}(\lambda _{0}))^{2}\mathrm {d}\varOmega + \int _{\varOmega }|\nabla \mathbf{n}(\lambda )|\mathrm {d}\varOmega , \end{aligned}$$

where $\delta$ is a regularization parameter and the unit normal vector field $\mathbf{n}$ is given by

3 $$\begin{aligned} \mathbf{n}(\lambda ) = \frac{\nabla \lambda }{|\nabla \lambda |_{\epsilon }} = \left( \frac{\lambda _{x}}{|\nabla \lambda |_{\epsilon }}, \frac{\lambda _{y}}{|\nabla \lambda |_{\epsilon }} \right) . \end{aligned}$$

In the second step, the surface $\lambda$ is found that fits the obtained smoothed normal vectors $\mathbf{n}(\lambda )$. The second step was defined as the *surface fitting* problem and it is performed with the following minimization problem:

4 $$\begin{aligned} \min _{\lambda }\frac{\gamma }{2}\int _{\varOmega }(\lambda -\lambda _{0})^{2}\mathrm {d}\varOmega + \int _{\varOmega } (|\nabla \lambda |_{\epsilon } - \nabla \lambda \cdot \mathbf{n}(\lambda ))\mathrm {d}\varOmega \end{aligned}$$

Since the minimization of (4) using noisy normal vectors $\mathbf{n}(\lambda _{0})$ will lead to a perturbed recovery, there has been a lot of research dedicated to the regularization of the normal vector field [12–15] or the tangential one [16]. The regularized normal vector field in the surface fitting step (4) can potentially improve image quality and remove the staircase effect of the lower order TV minimization methods (1). Note that when $\nabla \lambda \cdot \mathbf{n}(\lambda ) = 0$, the functional (4) becomes the classical TV minimization problem (1), on the other hand, when $\nabla \lambda \cdot \mathbf{n}(\lambda ) = |\nabla \lambda |_{\epsilon }$ the smoothing term disappears and the data fidelity term is in full force. Therefore the model (4) encourages the data fidelity term when $\nabla \lambda \Vert \mathbf{n}(\lambda )$ (structurally valuable regions, such as image boundaries) and more smoothing when $\nabla \lambda \bot \mathbf{n}(\lambda )$ (uniform regions).

If $\theta$ is an angle between $\nabla \lambda$ and $\mathbf{n}(\lambda )$, then one can rewrite the right hand side term in (4) as:

5 $$\begin{aligned} \int _{\varOmega }(|\nabla \lambda |_{\epsilon } - \nabla \lambda \cdot \mathbf{n}(\lambda ))\mathrm {d}\varOmega = \int _{\varOmega }|\nabla \lambda |_{\epsilon } (1 - \cos \theta )\mathrm {d}\varOmega . \end{aligned}$$

It is now evident that the smoothing term (5) approaches zero when $\theta \rightarrow 0$ (vectors parallel) and when $\theta \rightarrow \pi /2$ (vectors perpendicular) the weight of the TV penalty increases.

### Embedding Structural Information into the Surface Fitting Step

In this section we will show how one can embed supplementary information into the minimization term (4). Consider the following problem:

6 $$\begin{aligned} \lambda _{0} = \mathrm {A}\lambda + \eta , \end{aligned}$$

where $\lambda$ is an image we would like to recover from its noisy ($\eta$ noise component) and blurred representation $\lambda _{0}$, the forward operator $\mathrm {A}$ implements discrete convolution (for our problem it is isotropic blurring). The supplementary image $\mu$ is given as a reference image. The images can differ in intensity levels, geometry, spatial resolution and signal to noise ratio (SNR). The main goal is to recover (denoise and deblur) $\lambda$ using only the structural information of $\mu$, while preserving the salient features in $\lambda$.

One can substitute the normal vector fields of the reference $\mathbf{n}(\mu ) = \frac{\nabla \mu }{|\nabla \mu |_{\epsilon }}$ directly into (4), resulting in the regularized deblurring problem:

7 $$\begin{aligned} \min _{\lambda }\frac{\gamma }{2}\int _{\varOmega }(\mathrm {A}\lambda -\lambda _{0})^{2}\mathrm {d}\varOmega + \int _{\varOmega } (|\nabla \lambda |_{\epsilon } - \nabla \lambda \cdot \mathbf{n}(\mu ))\mathrm {d}\varOmega . \end{aligned}$$

However, this model assumes that the gradients of $\lambda$ are parallel to $\mu$ in every $(x,y)$. This is not a valid assumption for the reconstructed multimodal datasets where the gradient orientations can differ between acquired images. Following this observation, we believe that only parallel (or almost parallel) gradients of $\lambda$ and $\mu$ can be modified with additional information from $\mu$.

**Fig. 1:**
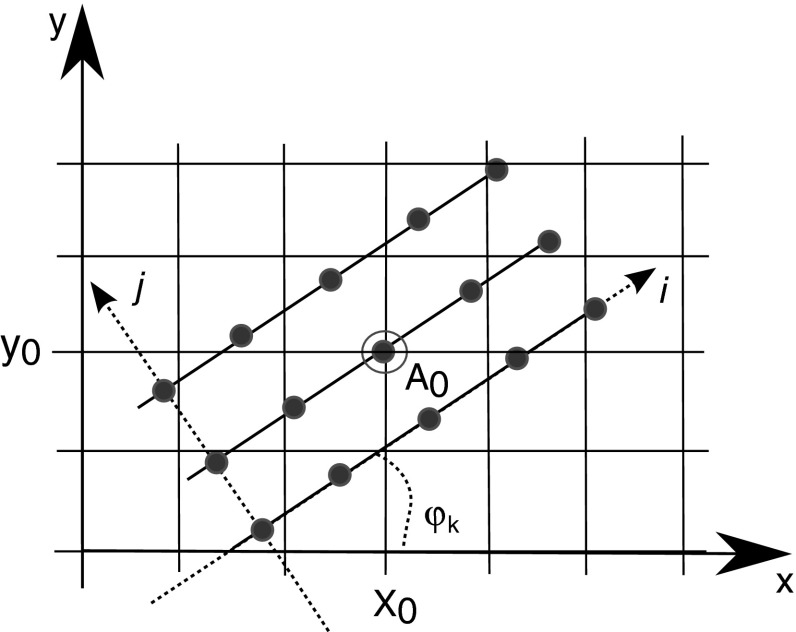
Example of an IRON symmetric network used to find orientation for the point $A_{0}(x_{0},y_{0})$ with $L=3$ *lines* and $p=5$ *points* per *line*

To identify how gradient orientations for $\lambda$ and $\mu$ are related to each other, one has to find an angle between $\nabla \lambda$ and $\nabla \mu$. In this paper we use an isotropic and recursive oriented network (IRON) to identify the gradient orientations [17]. This method has proven to be more stable to noise than derivative based approaches due to its non-local nature (see Fig. 1).

The computation of IRON for any image at a given location $(x_{0},y_{0})$ requires computation of the variance along the lines of the network (see Fig. 1). For each angle $\varphi _{k}, \ k = 1,\ldots , K$ the variance of the network is calculated as:

8 $$\begin{aligned} D(x,y,\varphi _{k}) = \frac{1}{L}\sum _{j}\left( \frac{1}{p}\sum _{i}(v_{i,j}^{2}) - \left( \frac{1}{p} \sum _{i}v_{i,j} \right) ^{2} \right) , \end{aligned}$$

here $v_{i,j}$ refers to the interpolated gray level at location $(i,j)$ on the network. The network consists of $L$ lines and $p$ points, for our experiments we take $L=3,p=5$. To obtain the desired texture orientation value one has to find a minimum (where global relates to one orientation and local to multiple) of the variance $D(x,y,\varphi _{k})$ for a given location $(x_{0},y_{0})$ for $k = 1,\ldots , K$ orientations. The number of orientations $K$ to be tested is defined by the application, in this work we used $K=16$, which is sufficient for our task. For the detailed description of the IRON method we refer the reader to [17], in our implementation we used the image rotation technique.

We define the texture orientations estimated with IRON method for images $\lambda$ and $\mu$ as $\varphi _{\lambda }(K)$ and $\varphi _{\mu }(K)$ respectively. Let $\varphi _{\lambda \mu }(K)$ be the angle between $\varphi _{\lambda }(K)$ and $\varphi _{\mu }(K)$ for $K$ orientations:

9 $$\begin{aligned} \varphi _{\lambda \mu }(K) = \varphi _{\lambda }(K) - \varphi _{\mu }(K); \ \varphi _{\lambda \mu }(K) \in [-\pi , \pi ]. \end{aligned}$$

Here we introduce an *orientation matching* measure which shows how gradient orientations are aligned with each other for $\lambda$ and $\mu$:

10 $$\begin{aligned} \varPhi (\varphi _{\lambda \mu }(K)) = 1 - \cos ^{2}(\varphi _{\lambda \mu }(K)), \end{aligned}$$

when $\varPhi (\varphi _{\lambda \mu }(K))\rightarrow 0$ the normal vectors tend to be parallel $\mathbf{n}(\lambda ) \Vert \mathbf{n}(\mu )$ or $\nabla \lambda \Vert \mathbf{n}(\mu )$.

In this paper, we say that the gradients of $\lambda$ and $\mu$ are parallel when $\varPhi (\varphi _{\lambda \mu }(K)) < T$, when $T$ is a small constant. Then the image recovery of $\lambda$ using $\mathbf{n}(\mu )$ can be written as:

11 $$\begin{aligned} \min _{\int _{\varOmega }(\mathrm {A}\lambda -\lambda _{0})^{2}\mathrm {d}\varOmega = \sigma ^{2}} \left\{ \begin{array}{ll} \int _{\varOmega }(|\nabla \lambda |_{\epsilon } - \nabla \lambda \cdot \mathbf{n}(\mu ))\mathrm {d}\varOmega &{} \quad \hbox {if } \varPhi (\varphi _{\lambda \mu }(K)) < T\\ \int _{\varOmega }|\nabla \lambda |_{\epsilon }\mathrm {d}\varOmega &{} \quad \hbox {else} \end{array} \right. \end{aligned}$$

The problem expressed in (11) describes the standard TV minimization (no prior information about the supplementary image used) for the areas where gradients $\lambda$ and $\mu$ are not parallel. The strong prior knowledge (direction of smoothing) is embedded when the gradients tend to be parallel.

The term $(|\nabla \lambda |_{\epsilon } - \nabla \lambda \cdot \mathbf{n}(\mu ))$ in (11) shares some similarities with the recently proposed model for nonlinear processing of color images [18], expressed by:

12 $$\begin{aligned} |\nabla \mu ||\nabla \lambda | - \mathrm {abs}(\nabla \mu \cdot \nabla \lambda ). \end{aligned}$$

This model measures the degree of level sets $\lambda ,\mu$ being parallel to each other and also depends on the gradient magnitudes of both images. In contrast to (12) we remove the dependency on the magnitude of the gradient for image $\mu$ in our model (11). Additionally, we would argue that in finding gradient orientations the IRON technique is more robust to noise than the derivative based techniques used in minimization of (12). However, a possible drawback of our method (11) is a binary decision making approach for the use of supplementary information (no linear combinations of vectors are taken). Here we do not compare the model expressed in (12) with the proposed one (11), but it is a subject of future research.

### Discretization of the Proposed Model

The optimality conditions for the saddle points of (11) are (considering only the upper part):

13 $$\begin{aligned}&-\nabla \cdot \left( \frac{\nabla \lambda }{|\nabla \lambda |_{\epsilon }} - \mathbf{n}(\mu ) \right) + \gamma (\mathrm {A}^{*}(\mathrm {A}\lambda - \lambda _{0})) = 0 \ \ \mathrm {in} \ \varOmega \end{aligned}$$

14 $$\begin{aligned}&\left( \frac{\nabla \lambda }{|\nabla \lambda |_{\epsilon }} - \mathbf{n}(\mu ) \right) \cdot \eta = 0 \ \ \mathrm {on} \ \partial \varOmega \end{aligned}$$

In (14), $\eta$ is the outwards unit normal vector on the boundary $\partial \varOmega$.

By introducing a time variable $t$ one can write (13) as an evolution equation:

15 $$\begin{aligned} \lambda _{t} = \nabla \cdot \left( \frac{\nabla \lambda }{|\nabla \lambda |_{\epsilon }} - \mathbf{n}(\mu ) \right) - \gamma (\mathrm {A}^{*}(\mathrm {A}\lambda - \lambda _{0})). \end{aligned}$$

For numerical implementation we use the notation of forward and backward differences: $\Delta _{\mp }^{x}\lambda _{i,j} = \mp (\lambda _{i \mp 1,j} - \lambda _{i,j})$ and $\Delta _{\mp }^{y}\lambda _{i,j} = \mp (\lambda _{i,j \mp 1} - \lambda _{i,j})$. We use an explicit scheme [11] to discretize (15) as:

16 $$\begin{aligned} \lambda _{i,j}^{n+1}&= \lambda _{i,j}^{n} + \Delta t \left[ \Delta _{-}^{x}\left( \frac{\Delta _{+}^{x}\lambda _{i,j}^{n}}{\left[ \left( \Delta _{+}^{x}\lambda _{i,j}^{n}\right) ^{2}+ \left( m\left( \Delta _{+}^{y}\lambda _{i,j}^{n},\Delta _{-}^{y}\lambda _{i,j}^{n}\right) \right) ^{2} + \epsilon ^{2}\right] ^{1/2}} - u_{i,j}(\mu ) \right) \right. \nonumber \\&\quad \left. +\, \Delta _{-}^{y}\left( \frac{\Delta _{+}^{y}\lambda _{i,j}^{n}}{\left[ \left( \Delta _{+}^{y}\lambda _{i,j}^{n}\right) ^{2} +\left( m\left( \Delta _{+}^{x}\lambda _{i,j}^{n},\Delta _{-}^{x}\lambda _{i,j}^{n}\right) \right) ^{2} + \epsilon ^{2}\right] ^{1/2}} - v_{i,j}(\mu ) \right) - \gamma (\widehat{\lambda }_{i,j}) \right] , \end{aligned}$$

where $u(\mu ) = \frac{\mu _{x}}{|\nabla \mu |_{\epsilon }}$, $v(\mu ) = \frac{\mu _{y}}{|\nabla \mu |_{\epsilon }}$, $\widehat{\lambda } = \mathrm {A}^{*}(\mathrm {A}\lambda ^{n} - \lambda _{0})$ and $m(a,b) = \mathrm {minmod}(a,b) = (\frac{\mathrm {sign}\ a + \mathrm {sign}\ b}{2})\min (\mathrm {abs}(a), \mathrm {abs}(b))$. The parameter $\Delta t$ denotes the time discretization constant and is chosen to be small for explicit schemes $0 < \Delta t \le 0.25$.

The proposed algorithm (11) to recover $\lambda$ having the supplementary image $\mu$ is given in Algorithm 1.

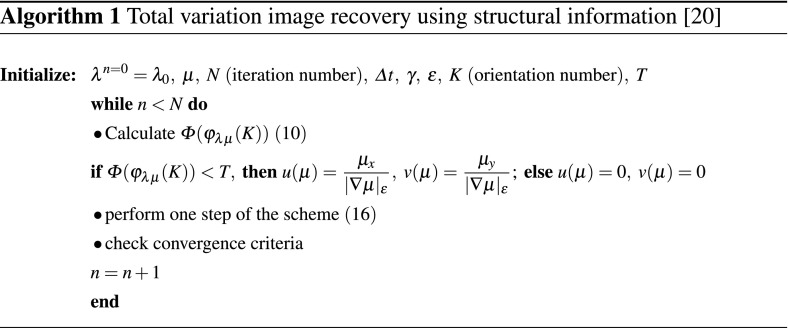



### Iterative Tomographic Reconstruction Using the Proposed Model

For tomographic reconstruction we consider a multimodal medical imaging set-up comprising functional (PET) and anatomical (X-ray CT) modalities [2]. Our aim is to reconstruct the unknown radiotracer distribution $\varvec{\lambda }$ having supplementary anatomical information $\varvec{\mu }$.

The image $\varvec{\lambda }\in \mathbb {R}^{N}$ which is an N-dimensional vector can be reconstructed from its projections (sinogram) $\varvec{g} \in \mathbb {R}^{M}$. For ET, $\varvec{g}$ follows a Poisson distribution and the count measurements can be written as:

17 $$\begin{aligned} g_{j} \sim \mathrm {Poisson}([\mathrm {P}\varvec{\lambda }]_{j}) \end{aligned}$$

where the projection or system matrix $\mathrm {P}:\mathbb {R}^{N} \rightarrow \mathbb {R}^{M}$ depends on the system design and the detector array geometry. In this work we do not account for the scatter effects, but the resolution of PET modality is simulated.

To reconstruct the image $\varvec{\lambda }$ from the measured data $\varvec{g}$, the following constrained cost function must be optimized:

18 $$\begin{aligned} \arg \min _{\varvec{\lambda }\ge 0} \mathbb {D}_{KL}(\varvec{g}; \mathrm {P}\varvec{\lambda }) + \beta \mathrm {R} (\varvec{\lambda }), \end{aligned}$$

where the Kullback–Leibler (KL) distance [21] is defined as:

19 $$\begin{aligned} \mathbb {D}_{KL}(\varvec{g}; \mathrm {P}\varvec{\lambda }) = \sum _{j}^{M} \left[ g\log \frac{g}{[\mathrm {P}\varvec{\lambda }]} - g + [\mathrm {P}\varvec{\lambda }] \right] _{j}, \end{aligned}$$

and the regularization term $\mathrm {R} (\varvec{\lambda })$ is controlled by the parameter $\beta$.

In this work, we consider three different regularization penalties, the first one is the traditional TV semi-norm

20 $$\begin{aligned} \mathrm {R_{1}} (\varvec{\lambda }) = \sum _{i}^{N}|\nabla \lambda _{i}|_{\epsilon }. \end{aligned}$$

The second and the third are anatomically driven functionals which depend on both $\lambda$ and $\mu$:

21 $$\begin{aligned} \mathrm {R_{2}} (\varvec{\lambda },\varvec{\mu }) = \left\{ \begin{array}{ll} \sum _{i}^{N}(|\nabla \lambda _{i}|_{\epsilon } - \nabla \lambda _{i} \cdot \mathbf{n}(\mu _{i})) &{} \quad \hbox {if } \,\varPhi _{i}(\varphi _{\lambda \mu }(K)) < T\\ \sum _{i}^{N}|\nabla \lambda _{i}|_{\epsilon } &{} \quad \text {else} \end{array} \right. \end{aligned}$$

and the penalty term is based on the Bowsher method (BM) [5, 10]:

22 $$\begin{aligned} \mathrm {R_{3}} (\varvec{\lambda ,\mu }) = \sum _{i}^{N}\sum _{k\in \aleph _{i}(\mu ,n_{0})} \rho _{\zeta }(\lambda _{i} -\lambda _{k}), \end{aligned}$$

where function $\rho$ is an edge preserving Huber function which approximates the $\ell _{1}$ norm similarly to the TV semi-norm [19]. The threshold $\zeta$ depends on $|\nabla \lambda |$ and needs to be carefully defined.

The penalty $\mathrm {R}_{3}$ performs smoothing between the central pixel $i$ and the nearest pixel $k$ in the local neighbourhood set $\aleph _{i}(\mu ,n_{0})$. The neighbourhood depends on $\mu$ alone and $n_{0}$ is a number of the most closest neighbours (normally 20–35 $\%$ of the total number of neighbours) of $i$ based on the smallest absolute differences $\mathrm {abs}(\mu _{i} - \mu _{k})$. The BM is based on the Gibbs assumption that the closest neighbours to the central pixel have the highest probability to be within one intensity class. One can use a simple absolute difference metric to find the most similar neighbours. This metric, however, is very sensitive to noise in images and we will demonstrate in numerical experiments later how a very low level of noise can significantly affect the quality of the recovered images. For more details on BM we refer the reader to [5, 10].

Similarly to [10] we write a nested forward-backward splitting iterative algorithm [22]:

23 $$\begin{aligned} \begin{array}{lcl} \varvec{\lambda }^{m+\frac{1}{2}} &{} = &{} \frac{\varvec{\lambda }^{m}}{\mathrm {P}^{*}{1}}\mathrm {P}^{*} \left( \frac{\varvec{g}}{\mathrm {P}\varvec{\lambda }^{m}} \right) , \ \ \textit{MLEM step} \\ \varvec{\lambda }^{m+1} &{} = &{} \mathrm {L}\left( \varvec{\lambda }^{m+\frac{1}{2}} \right) , \ \ \ \ \ \textit{denoising step} \end{array} \end{aligned}$$

Here the MLEM method solves the KL optimization sub-problem and $\mathrm {L}$ is an operator that performs a transition from $\varvec{\lambda }^{m+\frac{1}{2}}$ to $\varvec{\lambda }^{m+1}$ by minimizing the following function:

24 $$\begin{aligned} \varPsi (\varvec{\lambda }) = \frac{1}{2} \int _{\varOmega } \frac{\mathrm {P}^{*}{1}}{{\varvec{\lambda }}^{m}} \left( {\varvec{\lambda }} - \varvec{\lambda }^{m+\frac{1}{2}} \right) ^{2} +\, \beta \mathrm {R}(\varvec{\lambda }) \end{aligned}$$

The standard iterative gradient descent algorithm is used to optimize equation (24):

25 $$\begin{aligned} \varvec{\lambda }^{v+1} = \varvec{\lambda }^{v} - \Delta t\left[ \varPsi '(\varvec{\lambda }^{v})\right] . \end{aligned}$$

Using the proposed penalty (21) we present the following iterative reconstruction Algorithm 2 for tomographic reconstruction.

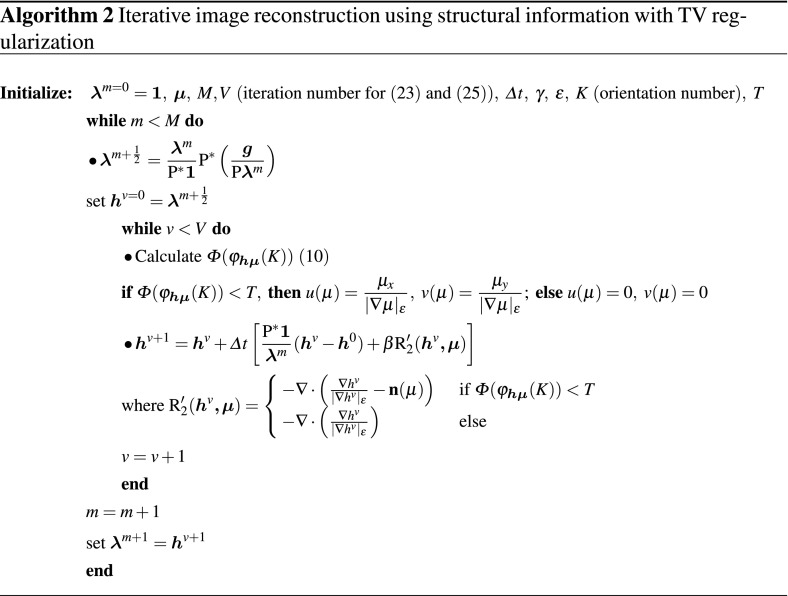



## Numerical Results

### Image Recovery Using Supplementary Information

**Fig. 2:**
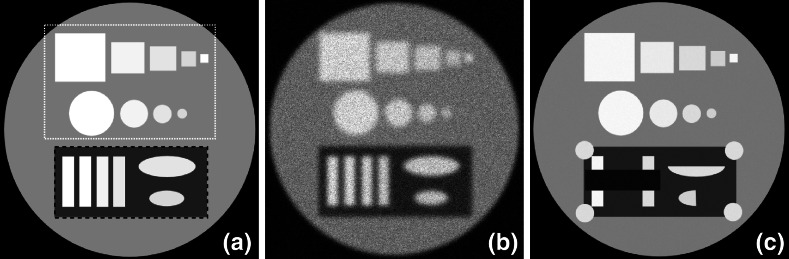
**a** Original image ${\lambda }$, the *upper box* (UB ROI) contains features that are present in the sharper reference image, while the *lower box* (LB ROI) contains partially correlated or structurally different features from the reference image, **b** isotropically blurred and noisy image ${\lambda }_{0}$ ($12\%$ of random noise), **c** the reference image ${\mu }$ with $0.05\%$ of noise. The main goal of the recovery process is to enhance resolution and SNR of $\lambda _{0}$ by using information from $\mu$ (**c**) without introducing any false features caused by uncorrelated edges in LB ROI

The aim of this experiment is to show that the proposed method is a flexible and easy-to-use tool for embedding supplementary structural information into the recovery process. We created two phantoms $\lambda$ and $\mu$ (see Fig. 2a, c, respectively) in a way that geometrically different structures were present in both images. Image $\lambda$ is significantly degraded by an isotropic blur (we use a Gaussian filter with $[15 \ 15]$ pixels kernel size and the standard deviation equal to 2.0) and noise with standard deviation of $12\%$ of the signal (see Fig. 2b). Image $\mu$ (the reference) is a less noisy dataset ($0.05\%$ of noise) with sharper features.

Potentially, $\mu$ can have different grey-scale intensity values, however, this will not impede the performance of the proposed method. Our aim here is to recover $\hat{\lambda }$ from $\lambda _{0}$ by using available structural information in $\mu$. Features of $\lambda$ which are geometrically correlated (common edges) with $\mu$ must be enhanced by information from $\mu$ during the recovery process, meanwhile the non correlated features must be preserved in $\hat{\lambda }$. Since some features in $\lambda$ are not correlated to features in $\mu$ (see LB ROI in Fig. 2a) can initiate false edges in the recovery of $\hat{\lambda }$, it is essential to restrict the use of supplementary information. This is a challenging task and failing to do so will result in severe artifacts in $\hat{\lambda }$.

For numerical experiments we use the gradient descent approach (25) to solve the least squares problem (LS) (6): $\lambda ^{n+1} = \lambda ^{n} - \Delta t(\mathrm {A}^{*}(\mathrm {A}\lambda ^{n} - \lambda _{0}))$. Different regularizes then applied to LS to stabilize solution against noise, such as TV (16) where $u,v = 0$, TV-Str method without orientation matching (7) and TV-Str with orientation matching (11) (see Algorithm 1). For the BM penalty we perform gradient descent iterations using the regularization term (22) in the Algorithm 3. For the image restoration experiment we provide the computer code which is implemented in C and Matlab languages and is available from the following link [20].

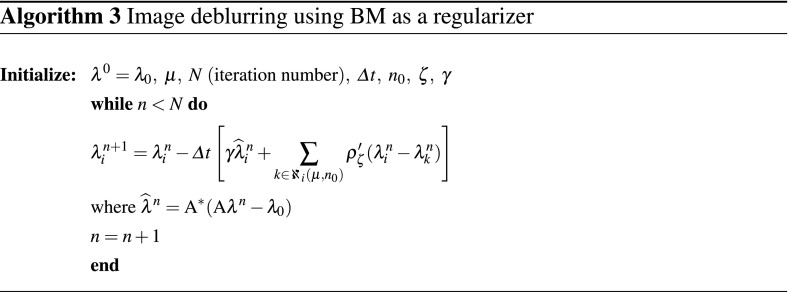



**Fig. 3:**
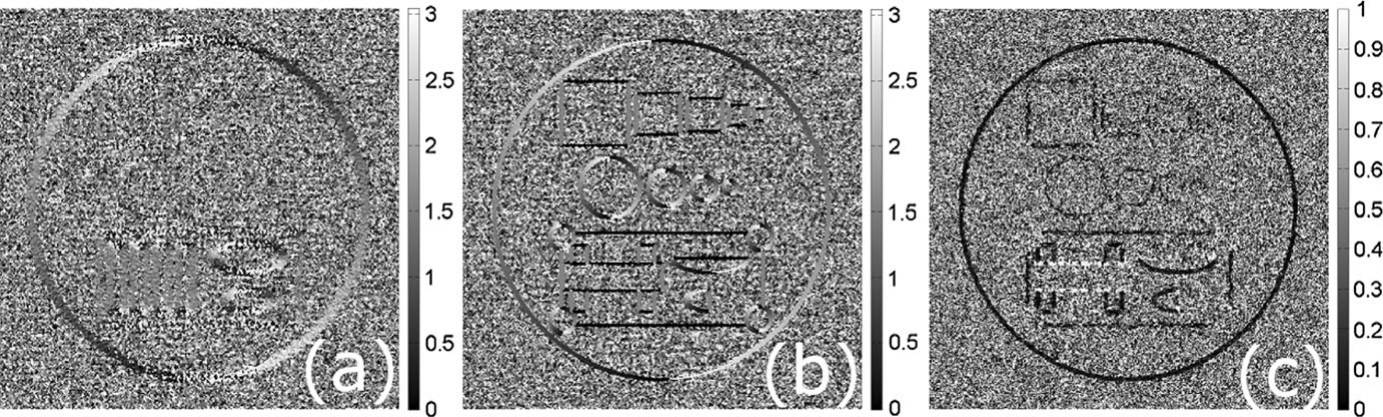
**a** The gradient orientation map $\varphi _{\lambda _{0}}(K)$ in radians for the degraded image, **b** the orientation map $\varphi _{\mu }(K)$ for the reference image, **c** the orientations matching measure $\varphi _{\lambda _{0}\mu }(K)$. Note that the lowest values of $\varphi _{\lambda _{0}\mu }(K)$ relate to the parallel gradients for $\lambda _{0}$ and $\mu$

In Fig. 3 we show the gradient orientations in radians calculated using the IRON method (8). In (a), one can see how angles $\varphi _{\lambda _{0}}(K)$ were estimated for the degraded image (first iteration of TV-Str Algorithm 1). In (b), the orientation map for the reference image is given and in (c) the orientation matching measure is calculated for $\lambda _{0}$ and $\mu$. One can see that even for the first iteration of the proposed TV-Str method the parallel gradients can be identified (low intensity values in (c)). During iterations of Algorithm 1 the orientation matching measure becomes more precise in identification of aligned features.

In this experiment all parameters were chosen empirically based on the known level of noise and the response from normalized mean square error (NMSE), given as:

26 $$\begin{aligned} NMSE(\hat{\lambda }, \lambda ) = \frac{\Vert \hat{\lambda } - \lambda \Vert _{2}}{\Vert \lambda \Vert _{2}}. \end{aligned}$$

In Table 1 we provide parameters which were used for this experiment.

**Table 1:** Parameters for the image restoration experiment

Method	$N$	$\gamma$	$\Delta t$	$\epsilon$	$K$	$T$	$n_{0}$	$\zeta$
(a) LS	7	–	0.1	–	–	–	–	–
(b) TV	200	50	0.002	$10^{-6}$	–	–	–	–
(c) BM	45	5	0.1	–	–	–	3	0.02
(d) TV-Str (no IRON)	50	30	0.002	$10^{-6}$	–	–	–	–
(e) TV-Str (IRON)	200	30	0.002	$10^{-6}$	16	0.03	–	–

See Algorithms 1 and 3

**Fig. 4:**
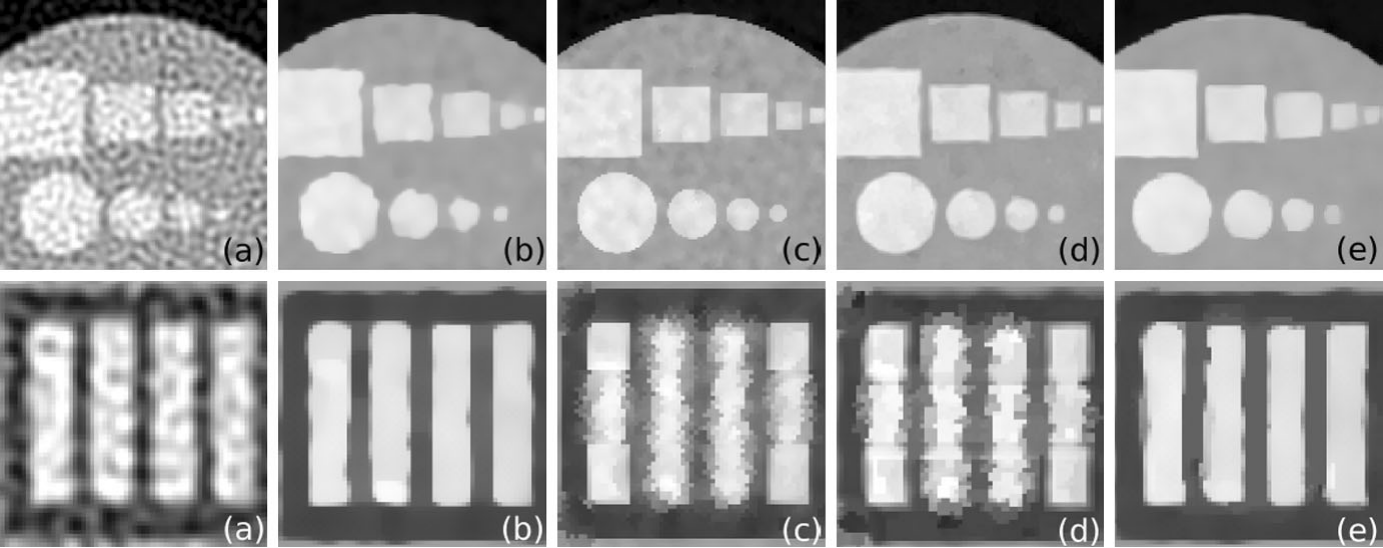
*Top row* shows a part of the recovered image $\hat{\lambda }$ in UB ROI using **a** LS, **b** TV (200 iterations), **c** BM (45 iterations), **d** TV-Str with no orientation matching step (7) (200 iterations), **e** TV-Str with orientation matching (11) (200 iterations). *Bottom row* shows a part of the image recovered in LB ROI. In the regions where information in $\mu$ is different from $\lambda$ (see **c**, **d**), strong artifacts have appeared and features are corrupted. In the proposed method (**e**) the major artifacts are successfully eliminated with the orientation matching technique (see Sect. 2.2). Parameters for this experiment are given in Table 1 and final errors are shown in Table 2

**Fig. 5:**
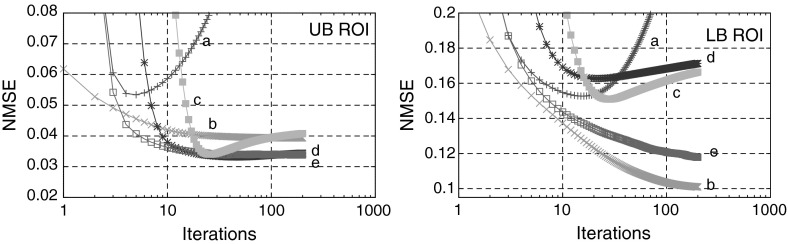
The plots of NMSE with respect to iteration number (logarithmic scale) for *a* LS, *b* TV , *c* BM, *d* TV-Str with no orientation matching step (7), *e* TV-Str with orientation matching (11). Note how the methods behave differently for the different ROIs. The parameters for this experiment are given in Table 1

In Fig. 4 (top) we consider the UB ROI where all features in $\lambda$ are ideally aligned with $\mu$. The LS method (a) fails to recover $\lambda$ due to strong influence of noise in the data. However, using TV regularization (b) one can remove noise and substantially improve resolution. The deblurring effect of the recovered images using the supplementary information can be clearly seen for the BM (c) and TV-Str methods (d, e). The convergence behaviour of the compared algorithms for the UB ROI can be seen in Fig. 5 (left). One can notice that the algorithms which use supplementary information give the smallest NMSE error (the BM (c) should be stopped earlier to avoid divergence). The TV-Str method without orientation matching (d) has almost the same error as TV-Str with IRON matching (e) (see Table 2)

**Table 2:** NMSE values for LS, TV, BM, TV-Str (no IRON) and TV-Str (IRON) methods

ROI	$\lambda _{0}$	LS	TV	BM	TV-Str (no IRON)	TV-Str (IRON)
UB	0.14	0.054	0.039	0.034	0.035	0.034
LB	0.24	0.155	0.10	0.152	0.16	0.115

.

In Fig. 4 (bottom) we consider the LB ROI where some features are aligned with each other and some are completely different. To demonstrate artifacts induced by the methods which use structural information (BM, TV-Str) we show the zoomed region of LB ROI. Very strong artifacts (horizontal lines) are visible using the BM and TV-Str without orientation matching (c, d). Both the BM and TV-Str without orientation matching deliver a high value of bias (see Fig. 5 (right)) for this ROI (the BM should be stopped prematurely). The proposed method with IRON orientation matching (e) delivers an image almost free of artifacts. According to the plots in Fig. 5 (LB ROI) the proposed method has slightly higher level of error than TV. In the presence of a high level of noise it is problematic to identify orientation of the gradient exactly.

In Table 2, the NMSE values for the methods are provided. The proposed method with orientation matching gives the best values for UB and competitive results for LB ROIs.

### Tomographic Image Reconstruction Using the TV-Str Method

**Fig. 6:**
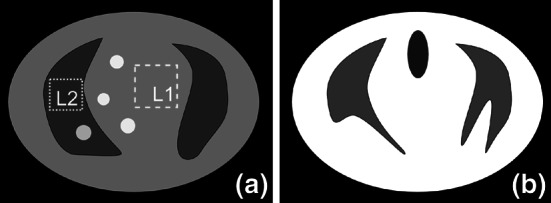
**a** The 2D ideal and noiseless functional phantom with several lesions. The background ROIs (*square boxes*) are used to calculate SNR (28) for the reconstructed lesion ROIs, **b** the reference (anatomical) image with correlated and uncorrelated features with the functional phantom

To further investigate the applicability of the proposed method we model a multimodal tomographic reconstruction problem. Our aim is to reconstruct a synthetic thorax phantom (see Fig. 6a) with supplementary information given in image (b). The functional (a) and anatomical (b) phantoms were chosen to be structurally different to examine the problem of misaligned features. Several lesions were added to the functional phantom which are absent from the reference phantom.

Each projection was generated with a strip kernel [23] using the higher resolution version of the phantom ($600 \times 600$ isotropic pixel grid). Reconstructions were calculated on a lower $200 \times 200$ isotropic pixel grid with a linear projection model thus avoiding the “inverse crime” of generating the data with the same model as the model that is used for calculating the reconstruction [24]. The pixel size was chosen to be 4 mm and the characteristic blur associated with the PET system was modelled by convolving each projection with a Gaussian kernel (FWHM = 5 mm) [3]. The resolution was not modelled in the reconstruction. Poisson distributed noise ($W = 30$ realizations) was applied to the projection data. The total number of counts was 100K per sinogram. Scatter was not simulated in this study. The number of acquisition angles was set to 400.

We compare the selected methods: MLEM (23) (upper step only), MLEM with TV penalty term (20), MLEM with TV-Str (21) and MLEM with BM (22). Since we perform the MLEM step similarly for every penalty function we reduce our notation for reconstruction methods with penalties to TV, BM, and TV-Str. Note that the TV-Str method here is the proposed method with orientation matching technique, without this step the proposed method can impose bias on the solution as shown earlier (see Sect. 3.1).

In order to compare algorithms we use the following quantitative measures: normalized absolute deviation (NAD), SNR and ROI variability.

The NAD between true activity ${\lambda }$ and the estimated $\hat{\lambda }$, over a ROI, is defined as:

27 $$\begin{aligned} \mathrm {NAD}(\hat{{\lambda }},{\lambda }) = \frac{1}{W}\sum _{w = 1}^{W} \left( \frac{\sum _{j\in ROI}|\hat{\lambda }_{w,j} - \lambda _{w,j}|}{\sum _{j\in ROI}\lambda _{w,j}}\right) \times 100, \end{aligned}$$

where $W$ is a number of noise realizations.

The SNR is defined as:

28 $$\begin{aligned} \mathrm {SNR}(\hat{\lambda }) = \frac{\frac{1}{W} \sum _{w=1}^{W}\left( \overline{\hat{\lambda }}^{ROI}_{w} - \overline{\hat{\lambda }}^{B}_{w}\right) }{\frac{1}{NB}\sum _{j\in B}\sigma _{j}^{W}}, \end{aligned}$$

where $\overline{\hat{\lambda }}^{ROI}$ and $\overline{\hat{\lambda }}^{B}$ is the average of counts within the ROI and the background, respectively. $NB$ is the total number of pixels within the background $B$ and $\sigma _{j}^{W}$ is the ensemble standard deviation of each pixel $j$ across all noise realizations $W$.

The ROI variability is defined as:

29 $$\begin{aligned} \mathrm {ROI \ variability}(\hat{\lambda }) = \frac{\frac{1}{ROI} \sum _{j\in B}\sigma _{j}^{W} }{\frac{1}{W}\sum _{w=1}^{W}\overline{\hat{\lambda }}^{ROI}_{w}}, \end{aligned}$$

Parameters for all methods were found empirically by referring to the best NAD-SNR values achievable. We did not perform a rigorous optimization for the parameter values, however certain conclusions based on the behaviour of each method can be made.

**Fig. 7:**
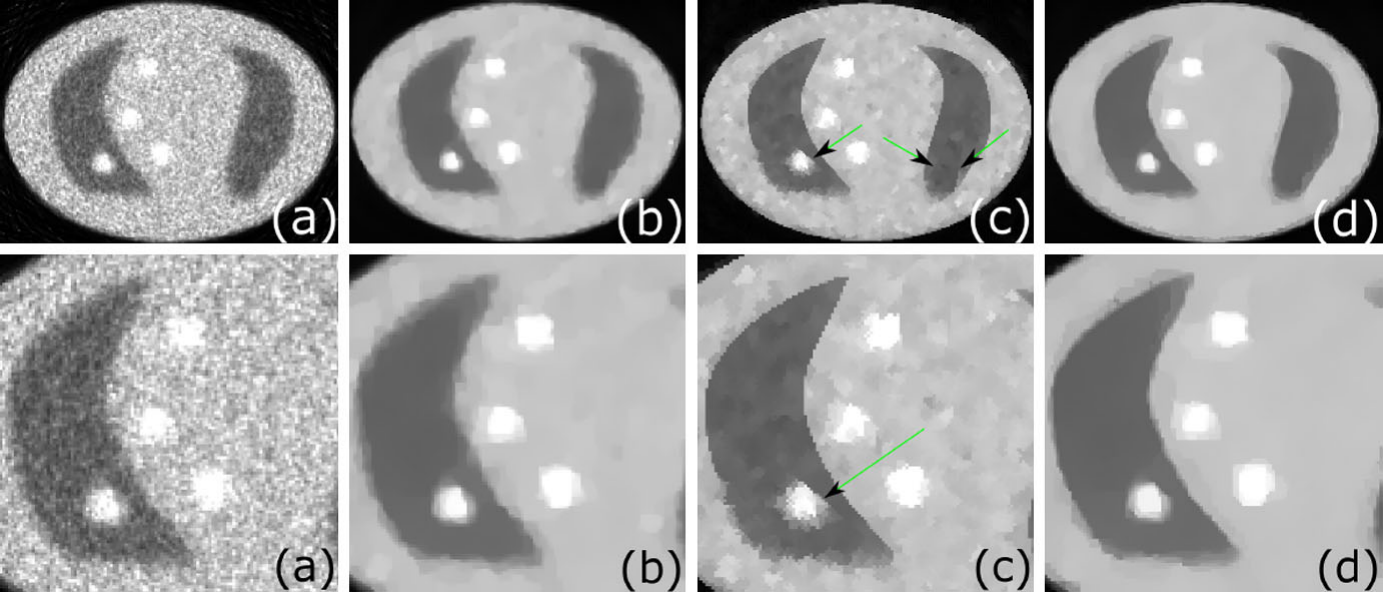
Reconstructions of the synthetic thorax using the primary and reference images, *top row* **a** MLEM reconstruction ($M = 50$), **b** TV ($V=15$), **c** BM ($V=2$) and **d** TV-Str ($V=15$); *bottom row* magnified regions. Note the improved resolution of TV-Str method, lowest level of noise and well preserved lesions. The BM gives strong artifacts (indicated by *arrows*) when edges of the functional phantom are misaligned with the reference image

**Fig. 8:**
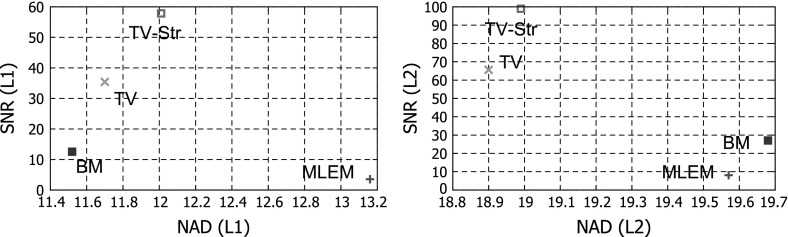
Bias-SNR values for L1 ROI (*left*) and L2 ROI (*right*). The proposed method TV-Str strongly outperforms all methods in SNR for lesion ROIs, however slightly higher in bias in comparison to TV reconstruction

**Fig. 9:**
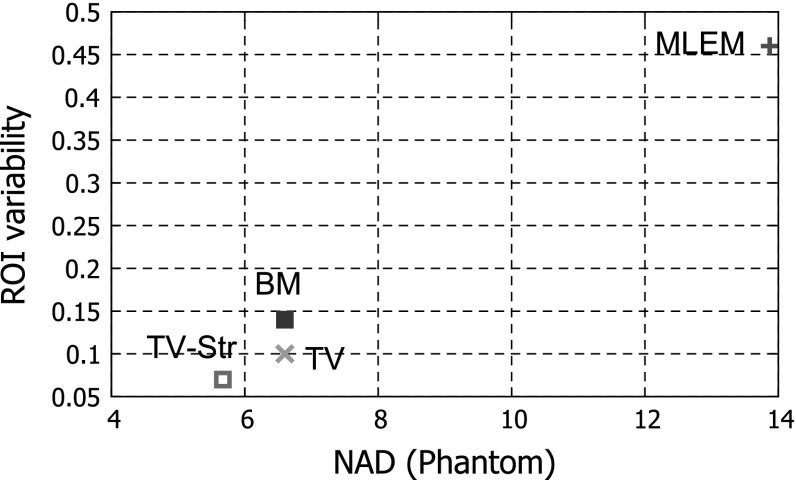
Bias-ROI variability for the whole phantom ROI. The proposed method TV-Str outperforms all methods in NAD and ROI variability for the whole phantom ROI

The MLEM algorithm gives an image with poor resolution and a high level of noise (see Fig. 7). To get a better reconstruction one needs to stop the iteration process prematurely [21], however in this case we run the MLEM algorithm for 50 iterations ($M = 50$). The quantitative analysis of lesion ROIs (see Fig. 8) and whole phantom (see Fig. 9) shows high values for NAD and low SNR. The NAD-SNR values are improved significantly with the use of TV regularization. The BM gives the lowest NAD for the L1 ROI (see Fig. 8) but quite a low SNR, for L2 ROI it shows low SNR as well. One can see the high variability level in reconstructed images on Fig. 7 as well as high value on Fig. 9. The proposed method TV-Str performs very similar to the TV penalty, but it also adds a significant amount of contrast to the edges which are considered to be common to both images. The NAD-SNR values for TV-Str (lesions ROI) show very competitive performance of the method providing higher values of SNR and NAD. For the whole phantom ROI the TV-Str method provides the best bias and the lowest ROI variability. On Fig. 7d one can see that lesions are well preserved (similar to TV) and no artifacts are visible.

## Discussion and Conclusion

In this work we have intentionally disregarded the first step of the problem (2), where one needs to regularize the normal or tangential vector fields. One can consider the case when two normal vector fields $\mathbf{n}(\lambda )$ and $\mathbf{n}(\mu )$ are minimized simultaneously resulting in the combined vector field $\mathbf{n}(\lambda ,\mu )$ which used in the surface fitting step (4). This is an interesting, yet challenging problem and it was partially examined previously using the combined diffusion tensors approach [10]. The choice between the orientations of two vector fields and the magnitude of the gradients is a complicated task which has a non-unique solution. In the future we will consider the problem of obtaining a smoothed joint vector field $\mathbf{n}(\lambda ,\mu )$.

We also used non-smoothed normals of $\mu$ in (4) and no strong artifacts appeared in the solution. However, if $\mu$ will be more noisy it is advisable to smooth it first before using in (4).

Notably, the functional (11) is non-differentiable due to discontinuity for $\lambda$ when $\varPhi = T$. The splitting techniques based on the proximity operators can deal with discontinuous penalty terms [22]. Another option is to modify functional (11) into convex and continuous combination which consists of TV and TV-Str terms in one regularization penalty.

Normally, using complex regularization terms in image reconstruction problems is discouraged due to difficulties in finding the minimizer for (18). The proposed TV-Str term with orientation matching is simple to use, however, the orientation matching step is computationally expensive. Faster and robust techniques to identify the aligned orientations for TV-Str can be strongly beneficial.

In this paper we have shown a novel approach for incorporating available additional information into TV filtering step. The resolution of features (common for various datasets) can be significantly improved while misaligned features can be recovered without strong artifacts. The proposed technique is robust to uncorrelated data since only parallel (or almost parallel) gradients are accepted for correction. The proposed functional can be used with many applications, such as, medical hybrid imaging, dynamic imaging (when pre-scan in higher resolution is available), image fusion etc.
